# Unlaid *Xenopus* eggs degrade by apoptosis in the genital tract

**DOI:** 10.1186/1471-2121-14-11

**Published:** 2013-03-03

**Authors:** Sho Iguchi, Tetsushi Iwasaki, Yasuo Fukami, Alexander A Tokmakov

**Affiliations:** 1 Graduate School of Science, Rokko dai 1-1, Nada, Kobe, 657-8501, Japan; 2 Research Center for Environmental Genomics, Kobe University, Rokko dai 1-1, Nada, Kobe, 657-8501, Japan

**Keywords:** Apoptosis, Unlaid eggs, Maturation, Ovulation, Meiotic exit, *Xenopus laevis*, Genital tract

## Abstract

**Background:**

In several species with external fertilization, including frogs, laid unfertilized eggs were found to die by apoptosis outside of the animal body. However, there is no apparent reason for the externally laid eggs to degrade by this process, considering that apoptosis developed as a mechanism to reduce the damaging effect of individual cell death to the whole organism.

**Results:**

Here, we demonstrate that a number of eggs are retained in the genital tract of the African clawed frog *Xenopus laevis* after gonadotropin-induced ovulation. The majority of these eggs exit meiotic arrest within 24 hours of hormone administration. Subsequently, post-meiotic eggs die in the frog genital tract by a well-defined apoptotic process. The hallmarks of egg degradation include prominent morphological changes, cytochrome *c* release, caspase activation, increase in ADP/ATP ratio, progressive intracellular acidification, egg swelling and all-out proteolysis of egg proteins. The sustained presence of post-apoptotic eggs in the genital tract of ageing frogs evidenced age-associated worsening of apoptotic clearance.

**Conclusions:**

The direct observation of egg degradation in the *Xenopus* genital tract provides a clue to the physiological relevance of frog egg apoptosis. It works to eliminate the mature unlaid eggs retained in the animal body after ovulation. Our findings establish egg apoptosis as a major physiological process accompanying ovulation in frogs.

## Background

In vertebrates, immature fertilization incompetent oocytes are naturally arrested in the prophase of the first meiotic division with the intact nuclear envelope and partially decondensed chromatin. During the steroid hormone-induced maturation oocytes progress through the meiotic cell cycle and arrest again in the metaphase of the second meiotic division. High activities of the key meiotic regulators, maturation promoting factor (MPF, a complex of cyclin B and Cdk1 kinase) and cytostatic factor (CSF, including Mos protein kinase and activated MAP kinase) have been established to maintain metaphase II arrest in mature eggs [1-4]. Fertilization causes the release of calcium from intracellular stores and egg activation. Then, calcium-dependent degradation of mitotic cyclins and Mos occurs, leading to MPF and CSF inactivation, meiotic exit, entry into the mitotic cell cycle and embryonic development.

Unfertilized mature eggs have been found to undergo a time-dependent quality loss in the process, which is referred to as postovulatory oocyte deterioration [5,6]. Delayed egg fertilization results in the progressive decrease of fertilization success in different frog, fish and mammalian species [7-10]. Spontaneous activation of ovulated eggs has been implicated as a probable biochemical basis for the time-dependent decrease of the fertilization rate [11,12]. It is still unclear how spontaneous activation and meiotic exit are triggered in the ovulated matured eggs. In different mammalian species, such as mice, rats, humans etc., ovulated eggs were shown to die by apoptosis if they are not fertilized [5,13-15]. Notably, in these species with internal fertilization, apoptotic cell death serves to remove aged unfertilized eggs without a pronounced inflammatory response, thereby supporting optimal body function.

Recently, it was found that unfertilized starfish eggs naturally deposited outside of the animal body also die by apoptosis [16-19]. In this species, spontaneous egg activation and meiotic exit were shown to precede initiation of the apoptotic program. Calcium and MAPK were implicated in triggering the apoptotic process [18,19]. Apoptotic induction in starfish eggs was demonstrated to require spontaneous inactivation of MAPK followed by activation of p38MAPK [18]. These findings raised the question about the physiological relevance of apoptosis in the matured ovulated eggs laid outside of the animal body. Evidently, there is no necessity for the externally laid eggs to die by this process, considering that apoptosis originated as a mechanism to reduce the damaging effect of individual cell death to the whole organism.

Most recently, two research groups, including ours, reported that the unfertilized *Xenopus* eggs laid outside of the frog body die by a well-defined apoptotic process [20,21]. In this case too, meiotic exit was found to precede apoptosis [21]. Notably, before being laid, *Xenopus* eggs are released from the ovaries into the coelomic body cavity. Then, the eggs pass through the oviduct where they acquire multiple coating jelly layers. Mature ovulated eggs have been observed at different locations of the frog body during egg-laying in the breeding season [22]. Egg accumulation in the uterus, or ovisac, has been documented [22-24]. Taken together, these facts suggested that similarly to the laid unfertilized eggs, the ovulated mature eggs retained in the frog body might also degrade by apoptosis. Indeed, Pasquier et al. reported caspase activation in the ovulated unlaid eggs retained in the frog genital tract [20]. However, the occurrence of other apoptotic events, dynamics of egg degradation in the frog body and emergence of meiotic exit in these eggs have not been investigated.

In the present study, we report that a number of mature metaphase-arrested eggs can be found at different locations of the *Xenopus* female frog body during several days following hormone-induced ovulation. The largest population of ovulated eggs is retained in the genital tract. The majority of these eggs exit meiotic arrest within 24 hours after hormone administration. Following meiotic exit, the eggs die in the frog genital tract by a well-ordered apoptotic process. The characteristic features of this process include cytochrome *c* release, caspase 3 activation, increase in the intracellular ADP/ATP ratio and massive proteolysis of egg proteins. At the late stage of egg death, prominent intracellular acidification and egg swelling are evident. All of these events have previously been observed in the unfertilized *Xenopus* eggs laid outside of the animal body [21], suggesting that the same apoptotic program unfolds in both retained and deposited eggs.

## Results

### Retention of ovulated eggs in the frog body

**Figure 1:**
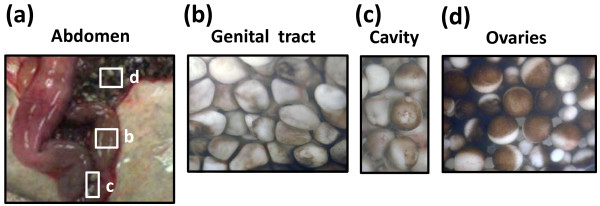
**Ovulated eggs are retained in the frog body at different locations.** The frog was dissected in 48 hours after hormonal stimulation **(a)** and the indicated fields of the abdomen **(b, c, d)** were observed microscopically.

It has been reported previously that ovulated frog eggs could be found in the *Xenopus* frog body in 40 hours after hCG-induced egg ovulation [20]. To further elaborate this finding, we investigated location, quantity and morphological appearance of the retained eggs. Ovulated eggs could be reproducibly detected at several locations of the frog body, such as the uterus, lower oviduct and peritoneal cavity at 48 hours after hCG injection (Figure 1a). By far the largest number of eggs was observed in the genital tract. These eggs were coated with the jelly layer acquired in the oviduct. Their appearance and coloring were abnormal, suggesting that egg degeneration took place at that time (Figure 1b). Although the ovisac contained the main stockpile of the eggs retained in the frog body after ovulation, a number of eggs could also be found in the upstream oviduct. Their morphology closely resembled that of the eggs retained in the ovisac.

In addition, a small number of single free-floating eggs could usually be seen in the coelomic body cavity. They were few and far between, so their exact number could not be reliably estimated. These eggs too had an altered pigmentation (Figure 1c), resembling that of the eggs found in the genital tract. In this study, we have not performed the detailed biochemical characterization of the free-floating coelomic eggs, as they represented only a minor fraction of all ovulated eggs retained in the frog body and they could hardly be subjected to the batch biochemical analysis due to the significant egg-to-egg variation.

In contrast to the previous report [20], we failed to detect the oocytes and eggs with an altered pigmentation in the ovaries of hCG-treated animals; they contained only healthy oocytes at different stages of differentiation (Figure 1d). Still, the presence of some abnormal oocytes/eggs in this location cannot be completely ruled out. They may be present as the single separated cells in different regions of the ovaries, making difficult their detection and en masse analysis.

These data demonstrate that a number of eggs are retained in the frog body after ovulation. Ordinarily, up to 10% of all ovulated eggs are not laid after hCG administration. The eggs retained in the genital tract represent the largest population of ovulated eggs in the frog body. Afterwards, the eggs isolated from the genital tract were subjected to the detailed morphological and biochemical analyses.

### Dynamics of egg degradation in the genital tract

**Figure 2:**
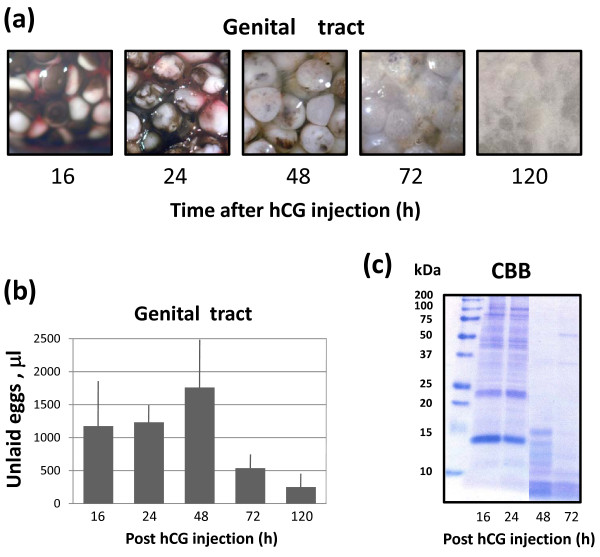
**Dynamics of egg degradation in the genital tract.** Changes in the morphology of eggs retained in the frog genital tract over five days are shown in **(a)**, the total volume of unlaid eggs in the uterus is presented in **(b)** and CBB staining of total egg proteins separated by SDS PAGE is shown in **(c)**.

Next, we investigated the dynamics of retention and morphological changes of the eggs found in the frog genital tract after ovulation. The appearance of these eggs changed significantly over time, indicating their progressive degradation. The eggs isolated from the genital tract at 16 hours after hCG administration displayed quite uniform morphology. They were coated with jelly layer and they had a prominent white spot in the animal hemisphere (Figure 2a). Afterwards, the characteristic changes in egg morphology occurred: the white spot became indistinguishable and the eggs acquired marble-like appearance. Subsequently, progressive decoloring of the pigment layer occured, individual eggs stuck together and formed a slurry where they finally became indistinguishable (Figure 2a).

Notably, the largest volume of ovulated eggs could be observed in the genital tract within the first 48 hours following hCG injection (Figure 2b). Later on by 72 hours, the volume of retained eggs decreased significantly and the eggs mostly disappeared from the genital tract by around 120 hours post hCG injection. In addition to the morphological changes, the robust proteolysis of various intracellular proteins has been observed in the unlaid eggs. The breakdown of high molecular weight proteins was evident in the eggs by the third day after hCG administration. It was accompanied by the accumulation of low molecular weight protein fragments and peptides (Figure 2c).

In sum, these results demonstrate that the progressive degradation of unlaid eggs occurs in the frog genital tract within several days following ovulation.

### Ovulated unlaid eggs spontaneously exit meiotic arrest

**Figure 3:**
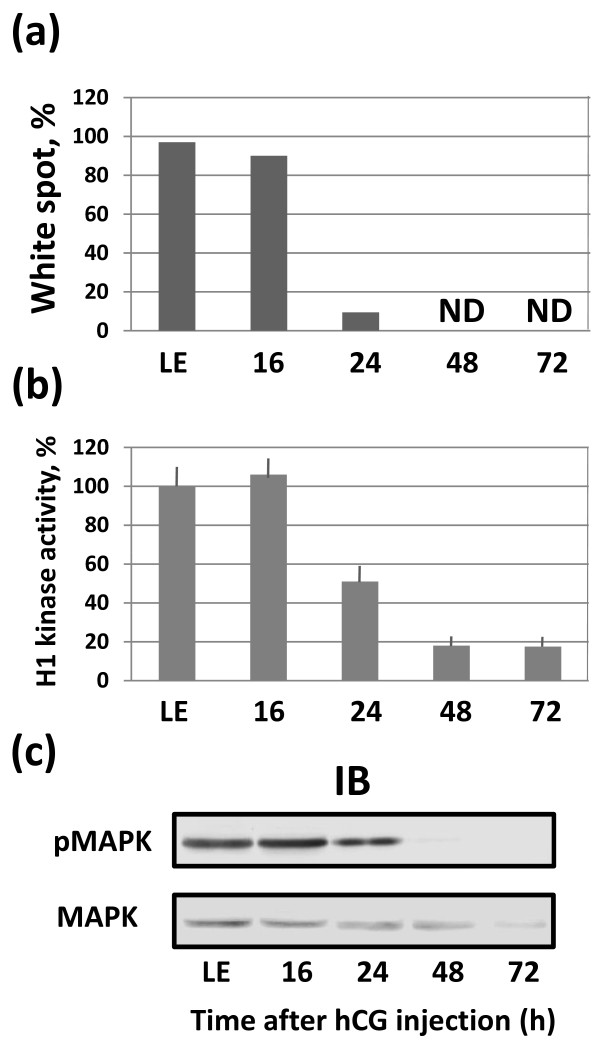
**Meiotic exit in the unlaid *Xenopus *eggs.** Ovulated eggs were isolated from the frog genital tract at different times (16-72 hours) after hCG administration. The percent of eggs with the white spot is shown in panel **(a)**, H1 kinase activity in the eggs is presented in panel **(b)**, MAPK contents and phosphorylation levels are shown in panel **(c).** LE in all panels refers to the eggs laid outside of frog body (laid eggs). Bars in panel **(b)** represent SD of three to four measurements of H1 kinase activity in the same biological material.

The morphological changes of the eggs retained in the frog body after ovulation resembled those observed in the naturally laid unfertilized *Xenopus* eggs. The laid eggs have previously been found to spontaneously exit meiotic arrest under various environmental conditions within 18 hours after deposition [21]. Therefore, next we investigated whether meiotic exit also occurred in the retained eggs. Initially, the eggs found in the *Xenopus* genital tract at 16 hours after hCG administration had a prominent white spot in the animal hemisphere, which is a characteristic feature of mature metaphase-arrested eggs (Figure 3a). Indeed, at that time the eggs were arrested in meiotic metaphase, as it can be judged by the high activity of the meiotic Cdk1 kinase and the presence of activated MAP kinase (Figure 3b,c). Afterwards, by 24 hours after hCG injection most eggs in the genital tract spontaneously exited metaphase arrest, as witnessed by disappearance of the white spot, decrease in Cdk1 activity and MAPK dephosphorylation (Figure 3a,b,c). Incomplete Cdk1 inactivation and MAPK dephosphorylation at 24 hours after hCG administration (Figure 3b,c) are consistent with the presence of some meiotically-arrested eggs at that time (Figure 3a), indicating rather poor synchronization of the meiotic exit between individual eggs. Notably, after MAPK dephosphorylation the physical degradation of the MAPK protein occurred in the ovulated unlaid eggs (Figure 3c) as a particular event of massive proteolysis ongoing in these cells (Figure 2c).

Altogether, these results show that similarly to the naturally laid unfertilized frog eggs, the ovulated unlaid eggs retained in the frog genital tract also exit meiotic arrest. The meiotic exit occurs in the majority of unlaid eggs by 24 hours after hCG administration and it is followed by robust degradation of egg proteins.

### Apoptotic degradation of unlaid *Xenopus* eggs

**Figure 4:**
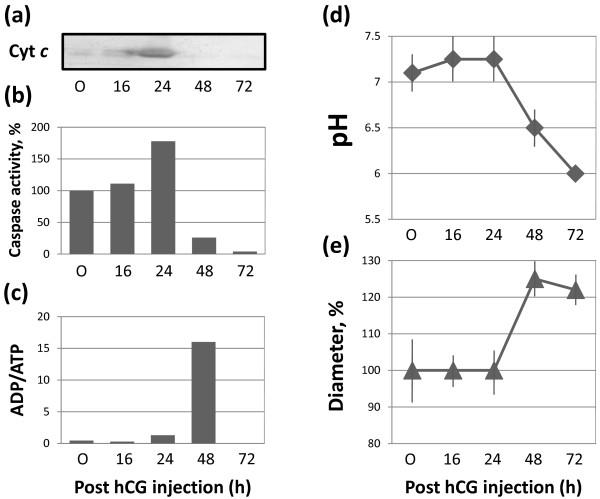
**Cell death events in the unlaid *Xenopus *eggs.** The features of classical apoptosis, such as cytochrome *c* release and caspase activation monitored over 72 hours after hormonal stimulation, are presented in panels **(a)** and **(b)**. The dynamics of ADP/ATP ratio, intracellular pH and egg diameter are shown in **(c), (d)** and **(e)**, respectively. In all panels, “O” refers to the oocytes isolated from the ovaries of ovulating animals. Bars in panel **(d)** represent the range of pH readings taken by two persons in double-blind trials and data in panel **(d)** are means + SD obtained by measurement of five to seven eggs.

Apoptosis has been reported to develop in the laid unfertilized *Xenopus* eggs after meiotic exit [21]. Next, we investigated whether apoptotic events also occured in the eggs retained in the frog body. We have found that the meiotically arrested unlaid eggs isolated from the genital tract at 16 hours after hCG administration exhibited low levels of cytoplasmic cytochrome *c*, basal caspase activity, low ADP/ATP ratio and near-neutral intracellular pH (Figure 4a-d). In fact, they were virtually indistinguishable by these parameters from the immature oocytes found in the ovaries of ovulating animals. The mechanisms of cellular osmotic homeostasis operated properly at that time, as it could be judged from the normal size of these eggs (Figure 4e). These data agree well with the previously reported results of *in vitro* maturation experiments [21]. However, a plethora of changes indicative of unfolding apoptotic process developed in the ovulated unlaid eggs after meiotic exit. At 24 hours after hormone-induced ovulation, the eggs released cytochrome *c* from mitochondria and they displayed significantly elevated levels of caspase 3 activity (Figure 4a,b). These phenomena represent the characteristic features of the classical mitochondria-mediated apoptotic process. Importantly, most of the eggs experienced meiotic exit by that time, as witnessed by disappearance of the white spot, decrease in Cdk1 activity and MAPK dephosphorylation (Figure 3a,b,c). The detailed consideration of causality between meiotic exit and apoptosis in these eggs is provided below in the “Discussion” section.

Subsequently, significant increase in the ADP/ATP ratio and prominent intracellular acidification were observed in the eggs by 48 hours after hCG administration (Figure 4c,d). Egg swelling at that time (Figure 4e) was indicative of membrane integrity loss, defining the terminal stage of cell death. The same features of cell death have been previously observed in the unfertilized *Xenopus* eggs laid outside of the animal body [21].

### Retention and degradation of ovulated eggs in ageing frogs

**Figure 5:**
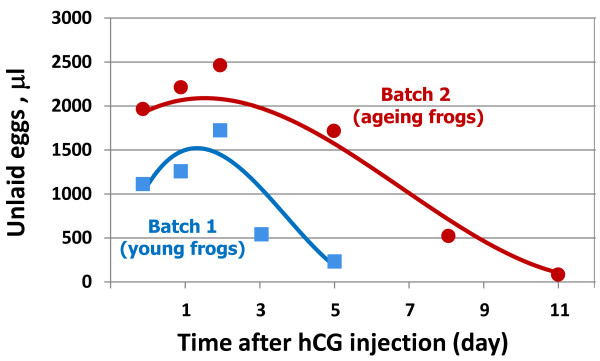
**Dynamics of egg retention in young and ageing frogs.** The total volume of unlaid eggs retained in the uteri of young (Batch 1) and ageing (Batch 2) frogs at different time after hCG administration is presented. The graphs were smoothed using Excel chart smoothing algorithm.

The animals used in the above experiments were two-year-old naïve *Xenopus* female adults. In addition, we also investigated the retention and degradation of ovulated eggs in the batch of ageing, 4- to 4.5-year old frogs. Over that time, the animals were kept in laboratory settings and they were repeatedly treated with hCG to induce egg ovulation. The physiological characteristics of frogs from the two studied batches are presented in Table 1. Importantly, the timing of egg deposition after hCG administration and the quality of deposited eggs were essentially the same in the two batches. The dynamics of egg retention in the genital tract of young and ageing frogs are shown in Figure 5. Notably, in comparison with the young frogs, the ageing animals retained a larger number of ovulated eggs for a longer period after hormonal stimulation. Nevertheless, the dynamics of morphological changes, meiotic exit and apoptosis were similar in the eggs obtained from the young and ageing frogs (Table 2). In addition, the largest volume of ovulated eggs was accumulated in the genital tract of both young and ageing frogs at about 48 hours after hCG injection (Figure 5, Table 2). Apparently, the sustained presence of ovulated eggs in the genital tract of ageing frogs is associated with the prolonged clearance of post-apoptotic eggs. These data provide evidence for the age-associated worsening of apoptotic egg clearance in *Xenopus* frogs.

**Table 1:** **Physiological characteristics of *Xenopus laevis *female frogs from two different batches**

**Batch**	**Young frogs**	**Ageing frogs**
**Parameter**		
**Age**	2 y.o.	4-4.5 y.o.
**Average body weight**	72 g	98 g
Start of egg laying*	within 11 h	within 12 h
Volume of laid eggs**	~ 15 ml	~ 20 ml
**Healthy eggs**	>90%	>90%
**Number of animals**	11	15

* time after hCG administration.

** packed volume of jelly layer-coated eggs.

**Table 2:** Timing of postovulatory events in the genital tracts of frogs from two different batches

**Batch**	**Young frogs**	**Ageing frogs**
**Parameter**		
**Morphological changes**	24 h	24 h
**Meiotic exit**	24- 48 h	24 -48 h
**Apoptosis**	within 48 h	within 48 h
**Maximal egg accumulation**	48 h	48 h
**Maximal volume of retained eggs**	1.7 ml	2.5 ml
**Presence in the genital tract**	5 days	11 days

## Discussion

We demonstrate here that a number of eggs are retained at different locations of the frog body over several days following hCG-induced egg ovulation. By far the biggest population of the unlaid eggs was found to reside in the frog genital tract, including the uterus and lower oviduct. The largest volume of ovulated eggs was retained in the genital tract during the first 48 hours following hCG injection and their volume significantly decreased by 72 hours (Figure 2b). A tendency (albeit with low statistical significance) has been observed to the increase of the ovisac volume by 48 hours after hCG administration (Figures 2, 5). It can possibly be attributed to at least two factors. One of them is egg swelling, which takes place by that time as a result of membrane integrity loss and osmotic homeostasis disruption (Figure 4e). In addition, some eggs are slow to get to the ovisac after ovulation. A number of these eggs could be detected in the oviduct on their way to the uterus early after ovulation. These eggs might finally reach the ovisac and accumulate there with some delay, thereby contributing to its volume.

Notably, a small number of eggs could also be detected in the frog coelomic cavity (Figure 1c). In frogs, it is established that ovulated eggs are released into the coelom before they reach the oviduct [22-26]. We have not investigated in detail the dynamics of morphological and biochemical changes of the coelomic eggs. However, the fact that the ovulated eggs of degenerative morphology were still present in the coelomic cavity at 48 hours after hCG administration strongly suggest their degradation at this location. Interestingly, a rare complication of ovarian hormonal stimulation, the ovarian hyperstimulation syndrome, has been described in gonadotropin-treated *Xenopus* frogs [27]. The coelomic cavity of the affected frogs was found to be filled with numerous free-floating and adherent degenerative oocytes. At present, their identity has not been disclosed and the mechanisms of their degeneration have not been established. However, considering their etiology, location and morphology, it is tempting to surmise that the abnormal oocytes observed in the frogs with the ovarian hyperstimulation syndrome may represent the ovulated post-meiotic apoptotic eggs described in this study. Additional experiments are necessary to validate this hypothesis.

We have reported previously that naturally laid unfertilized frog eggs spontaneously exit meiotic metaphase arrest under various environmental conditions and this meiotic exit is required for execution of the apoptotic program. This situation is reminiscent of the apoptotic induction in star fish eggs, where meiotic arrest effectively blocks apoptosis [16,18]. Our present study demonstrates that the frog eggs retained in the genital tract also exit meiotic arrest within 24 hours after hCG injection. These data agree well with the previously reported dynamics of meiotic exit in the naturally laid unfertilized *Xenopus* eggs, which were shown to exit meiotic arrest within 18 hours of their deposition [21].

In accordance with previous studies, we have found that apoptosis develops in the ovulated unlaid frog eggs after the meiotic exit. Indeed, the hallmark events of classical apoptosis, such as cytochrome c release and caspase activation, were observed in the eggs retained in the *Xenopus* genital tract at 24 hours after hCG-induced egg ovulation (Figure 4a,b). By that time, most of the unlaid eggs experienced meiotic exit, as it can be judged by disappearance of the white spot, decrease in Cdk1 activity and MAPK dephosphorylation (Figure 3). The presence of a minor fraction of meiotically-arrested eggs at that time (Figure 3) reveals rather poor synchronization of meiotic exit between the individual eggs, suggesting considerable heterogeinity of apoptotic response in the population of unlaid eggs. Previously, the single-cell analysis of deposited unfertilized frog eggs also evidenced a significant egg-to-egg variation of caspase activation [21]. It is highly improbably that apoptosis may develop in the minor fraction of meiotically-arrested eggs present in the genital tract at that time, considering that none of the investigated apoptotic events could be detected in the population of predominantly meiotic eggs at 16 hours after hCG administration. Moreover, in the laid unfertilized *Xenopus* eggs, meiotic exit was shown to be a prerequisite for execution of the apoptotic program, since (i) it always precedes apoptosis, (ii) apoptosis does not occur in the eggs maintaining high activity of MPF and CSF, and (iii) apoptosis is accelerated upon early meiotic exit [21]. In addition, it has been demonstrated that the metaphase-arrested cell-free extracts of *Xenopus* eggs are markedly refractory to apoptosis [28,29]. It was found that the MAPK pathway active in the metaphase egg extracts renders them resistant to apoptosis [29]. Anti-apoptotic role of Cdk1/cyclin B has also been established in the mitotic cells [30,31]. Additional analyses are necessary to explicitly address the issue of causality between meiotic exit and apoptosis in order to elaborate the emerging anti-apoptotic role of meiotic metaphase arrest.

Observation of egg degradation in the genital tract of gonadotropin-treated *Xenopus* frogs raises a question about the fate of naturally ovulated unlaid eggs. Notably, in laboratory settings, the frogs are stimulated to lay eggs by hCG injections. The hCG dose varies widely between 50 and 800 IU per animal [27]. This allows collecting sufficient quantities of mature eggs from healthy frogs. In the natural world too, ovulation in frogs is triggered by the somatic release of gonadotropins during the breeding season [32]. Considering that accumulation of mature eggs has been observed in the frog genital tract during that time [22-24], it is easy to conceive that the naturally ovulated unlaid eggs also degrade in the frog body by the apoptotic mechanism described in this study. However, this suggestion requires verification in prospective studies.

At present, it is unclear how the final elimination of egg remnants occurs from the genital tract. On the one hand, egg disposal outside of the frog body, in a way similar to laying off matured ovulated eggs, seems likely. However, it is arguable whether this kind of discarding can occur in the absence of a strong hormonal signal that induces egg ovulation and deposition. On the other hand, apoptotic cell death in the organism normally culminates in recognition and ingestion of dying cells by phagocytes. After apoptotic cell engulfment, macrophages activate tolerogenic pathways to prevent immune response against self-antigens [33]. Immune homeostasis is essential in maintaining apoptotic cell clearance [34]. In this connection, the sustained presence of post-apoptotic eggs in the genital tract of ageing frogs revealed in this study (Figure 5, Table 2) may be indicative of the age-associated decline in the immune system homeostasis. Presently, there is no data concerning the activity of the frog innate immune system in the postovulatory period. The final steps of the apoptotic egg clearance in the frog body still remain to be elucidated.

## Conclusions

**Figure 6:**
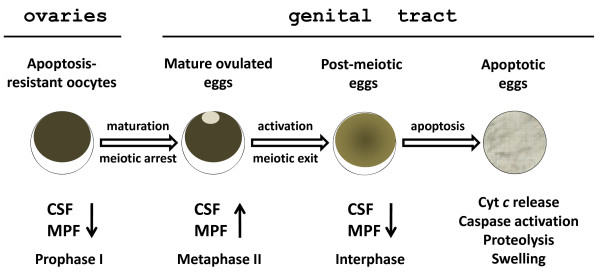
**Degradation of the unlaid *Xenopus *eggs.** In the ovaries, fully grown apoptosis-resistant oocytes are naturally arrested in prophase I with the low activity of CSF and MPF. After hormone-induced ovulation, a number of matured eggs now arrested at the metaphase II by the high activity of CSF and MPF are retained in the frog genital tract. They experience spontaneous meiotic exit and initiate a classical cytochrome c-mediated apoptotic program, accompanied by global proteolysis of intracellular proteins. Finally, the eggs completely deteriorate and lose their integrity inside the frog body.

The present study reveals that: i) a number of mature metaphase-arrested eggs are retained in the frog body after ovulation, ii) the majority of these eggs remain in the genital tract and degrade there within several days after hCG-induced ovulation, iii) in comparison with young frogs, ageing animals retain a larger number of ovulated eggs for a longer postovulatory period, iv) most of the retained eggs exit metaphase arrest spontaneously within 24 hours after hCG administration, v) the retained eggs die in the frog genital tract by a well-defined apoptotic process, which is characterized by cytochrome *c* release, caspase activation, increase in ADP/ATP ratio, progressive intracellular acidification, egg swelling and all-out proteolysis of intracellular proteins. These findings demonstrate that the process of ovulation in frogs is ordinarily accompanied by apoptosis of post-meiotic eggs in the genital tract, as summarized in Figure 6.

## Methods

### Materials

Female frogs of *Xenopus laevis* were purchased from Hamamatsu Seibutsu Kyozai (Hamamatsu, Japan). Human chorionic gonadotropin (hCG) was from Teikoku Zoki (Tokyo, Japan). Polyclonal anti-MAPK and anti-pMAPK antibodies were from Cell Signaling (Beverly, MA). Anti-cytochrome *c* antibody and alkaline phosphatase-conjugated goat polyclonal antibody against rabbit IgG were from Santa Cruz (Santa Cruz, CA). Microcon centrifugal filter devices (MWCO 100 kDa) were from Millipore (Billerica, MA). pH test paper for the intervals 5.4 – 7.0 and 0 – 14 was from Advantec Toyo Roshi (Tokyo, Japan) and Macherey-Nagel (Dueren, Germany). Fluorogenic caspase-3 substrate IV was from Calbiochem (La Jolla, CA) and fluorescent pH indicator BCECF was purchased from Wako (Tokyo, Japan). Bioluminescent ApoSENSOR ADP/ATP ratio assay kit was obtained from BioVision (Mountain View, CA) and protein assay kit was from Bio Rad (Hercules, CA). [γ-^32^P]ATP was obtained from Japan Radioisotope Association (Tokyo, Japan). Other chemicals were from Wako, Nacalai Tesque (Kyoto, Japan), or Sigma (St. Louis, MO).

### Animal treatment and egg isolation

Animal handling was carried out in accordance with the guidelines of the Kobe University Animal Experimentation Regulations. The study was approved by the Institutional Animal Care and Use Committee (permission number for animal facility agreement and authorization to manipulate animals KEN-12). The frogs were handled under strict laboratory rules. *Xenopus* female frogs were maintained in dichloride tap water at the ambient temperature (21-23°C). To induce ovulation, frogs were injected hypodermically with 500 IU per animal of hCG in the dorsal lymph sac. The frogs started to lay eggs in 11-12 hours after hormonal stimulation. Eggs retained after ovulation in the genital tract were isolated by frog dissection at different times after hCG injection. Before dissection, frogs were anesthetized on ice followed by rapid decapitation. Microscopic observations of egg morphology were carried out using a stereomicroscope (Leica S8APO). Egg images were acquired with the EC3 stereomicroscope digital color camera and processed with the Leica Application Suite LAS EZ V1.8.

### Biochemical analyses

For biochemical analyses, eggs were mildly dejellied with 1% cysteine. Caspase 3 activity assay, cytochrome *c* release assay, pH measurements, immunoblotting, determination of ADP/ATP ratio, protein assay and protein kinase assay were performed as detailed elsewhere [21,35].

### Other methods

Protein SDS PAGE and gel staining with Coomassie Brilliant Blue were carried out as described previously [36]. Egg diameter was determined by calculating the arithmetic mean of two perpendicular measurements taken in the microscopic egg image.

## Abbreviations

CSF: cytostatic factor; GVBD: germinal vesicle breakdown; hCG: human chorionic gonadotropin; MAPK: mitogen-activated protein kinase; MPF: maturation promoting factor

## Competing interests

The authors declare that they have no competing interests related to the manuscript.

## Authors’ contributions

AAT designed the research, performed the experiments and wrote the paper. SI performed the experiments and analyzed data. TI and YF contributed critical comments and suggestions. All authors read and approved the final manuscript.
